# Feasibility and potential efficacy of a guided internet- and mobile-based CBT for adolescents and young adults with chronic medical conditions and comorbid depression or anxiety symptoms (youthCOACH_CD_): a randomized controlled pilot trial

**DOI:** 10.1186/s12887-022-03134-3

**Published:** 2022-01-29

**Authors:** A. Geirhos, M. Domhardt, F. Lunkenheimer, S. Temming, R. W. Holl, K. Minden, P. Warschburger, T. Meissner, A. S. Mueller-Stierlin, H. Baumeister

**Affiliations:** 1 Department of Clinical Psychology and Psychotherapy, Institute of Psychology and Education, Ulm University, Ulm, Germany; 2 Faculty of Engineering, Computer Science and Psychology Institute of Psychology and Education, Department of Clinical Psychology and Psychotherapy, Ulm University, Lise-Meitner-Straße 16, 89081 Ulm, Germany; 3 Department of Pediatric Respiratory Medicine, Immunology and Critical Care Medicine, Charité Universitätsmedizin Berlin, Berlin, Germany; 4 Institute for Epidemiology and Medical Biometry, ZIBMT, Ulm University, Ulm, Germany; 5 Charité University Medicine Berlin, Berlin, Germany; 6 German Rheumatism Research Centre, Berlin, Germany; 7 Department of Psychology, Counseling Psychology, University of Potsdam, Potsdam, Germany; 8 Department of General, Paediatrics, Neonatology and Pediatric Cardiology, Medical Faculty, University Hospital Düsseldorf, Heinrich-Heine-University, Düsseldorf, Germany; 9 Department of Psychiatry and Psychotherapy II, BKH Günzburg, Ulm University, Günzburg, Germany

**Keywords:** Chronic medical condition, Depression, Anxiety, Internet- and mobile based intervention, Cognitive behavioral therapy, Randomized controlled pilot trial, Type 1 diabetes, Cystic fibrosis, Juvenile idiopathic arthritis

## Abstract

**Background:**

Adolescents and young adults (AYA) with a chronic medical condition show an increased risk for developing mental comorbidities compared to their healthy peers. Internet- and mobile-based cognitive behavioral therapy (iCBT) might be a low-threshold treatment to support affected AYA. In this randomized controlled pilot trial, the feasibility and potential efficacy of youthCOACH_CD_, an iCBT targeting symptoms of anxiety and depression in AYA with chronic medical conditions, was evaluated.

**Methods:**

A total of 30 AYA (*M*_age_ 16.13; *SD*= 2.34; 73% female), aged 12-21 years either suffering from cystic fibrosis, juvenile idiopathic arthritis or type 1 diabetes, were randomly assigned to either a guided version of the iCBT youthCOACH_CD_ (IG, *n*=15) or to a waitlist control group (CG, *n*=15), receiving an unguided version of the iCBT six months post-randomization. Participants of the IG and the CG were assessed before (t0), twelve weeks after (t1) and six months after (t2) randomization. Primary outcome was the feasibility of the iCBT. Different parameters of feasibility e.g. acceptance, client satisfaction or potential side effects were evaluated. First indications of the possible efficacy with regard to the primary efficacy outcome, the Patient Health Questionnaire Anxiety and Depression Scale, and further outcome variables were evaluated using linear regression models, adjusting for baseline values.

**Results:**

Regarding feasibility, intervention completion was 60%; intervention satisfaction (*M* = 25.42, *SD* = 5.85) and perceived therapeutic alliance (*M* = 2.83, *SD* = 1.25) were moderate and comparable to other iCBTs. No patterns emerged regarding subjective and objective negative side effects due to participation in youthCOACH_CD_. Estimates of potential efficacy showed between group differences, with a potential medium-term benefit of youthCOACH_CD_ (β = -0.55, 95%CI: -1.17; 0.07), but probably not short-term (β = 0.20, 95%CI: -0.47; 0.88).

**Conclusions:**

Our results point to the feasibility of youthCOACH_CD_ and the implementation of a future definitive randomized controlled trial addressing its effectiveness and cost-effectiveness. Due to the small sample size, conclusions are premature, however, further strategies to foster treatment adherence should be considered.

**Trial registration:**

The trial was registered at the WHO International Clinical Trials Registry Platform via the German Clinical Trials Register (ID: DRKS00016714, 25/03/2019).

**Supplementary Information:**

The online version contains supplementary material available at 10.1186/s12887-022-03134-3.

## Background

Worldwide, approximately 15-20% of adolescents and young adults (AYA) live with chronic medical conditions [1]. In recent decades the incidence rate increased [2]. Chronic conditions can impact the cognitive, physical, social and emotional development of afflicted AYA beyond the usual developmental challenges at this age [3]. In comparison to their healthy peers, young people with a chronic medical condition report elevated internalizing and externalizing behavior problems. Particularly, symptoms of anxiety and depression are increased in AYA with chronic conditions in comparison to their healthy peers [4, 5]. Comorbid mental health issues can have an important impact on AYAs’ quality of life, disease course, regime adherence and medical outcomes [6].

In adolescence, worsening in inborn or acquired disease state can be found. Therefore, research in clinical practice has been focused to improve clinical outcomes [7]. Nonetheless, the significant impact of mental comorbidities on clinical outcome parameters as well as quality of life highlights the need for a multidisciplinary approach [8]. Especially, as the prevalence of mental disorders generally increases in adolescence compared to childhood [9].

 Treatment guidelines recommend cognitive behavioral therapy (CBT) as first line approach for AYA with mental health issues (e.g. [10]). There is a growing evidence base reporting on the effectiveness of CBT for the prevention and treatment of anxiety and depression symptoms in AYA [11, 12]. Studies also suggest the effectiveness of CBT when specifically targeting AYA with chronic conditions and comorbid symptoms of anxiety and depression [13]. However, access to psychological and behavioral interventions is limited for many patients. Only a small proportion of AYA with chronic conditions in need of mental health services actually receives professional support [6, 14, 15]. In a Dutch web-survey of AYA with type 1 diabetes (T1D) only 28% of participants with symptoms of depression received psychological care [15]. Unmet health care needs highly impact AYAs’ physical and mental health outcomes in adulthood [16]. Reasons for this mental health care gap might be a lack of financial and personnel resources, structural reasons, such as healthcare systems, availability of resources, as well as personal reasons such as lack of problem awareness, lack of time, living in rural areas, fear of stigmatization and distrust towards mental health care [15–18].

As an opportunity to overcome some of the challenges associated with traditional face-to-face mental health care, internet- and mobile-based interventions (IMI) have been suggested. In the general population of AYA, IMI, especially based on CBT (iCBT), have been shown to be efficacious in reducing symptoms of anxiety and depression [19]. The evidence for utilizing IMI on AYA with chronic conditions points to their feasibility and effectiveness [20]. However, only a few studies focus on the efficacy of IMI targeting AYA with a chronic condition and comorbid mental health disorders. Meta-analyses include only a small number of studies focusing exclusively on internalizing symptoms. These studies showed limited methodological quality and indicate limited efficacy [21, 22]. Therefore, we aimed to develop a user-centered iCBT for AYA with a chronic medical condition and comorbid symptoms of anxiety and depression and evaluate its potential efficacy in an appropriate study design in the framework of the COACH project (Chronic conditions in adolescents: implementation and evaluation of patient-centered collaborative healthcare). The iCBT, called youthCOACH_CD_ (CD = Chronic conditions) is intended to provide an evidence-based low-threshold service for this target group. In addition to reducing symptoms of anxiety and depression, youthCOACH_CD_ aims to support AYA in dealing with further potential symptoms of mental disorders that are highly important in adolescence and when living with a chronic medical condition: potential symptoms of post-traumatic stress disorder due to (treatment of) the chronic condition [23]; risk-taking behaviors such as excessive alcohol consumption, often associated with symptoms of anxiety and depression [24, 25]; coping with the chronic condition; self-efficacy [26].

The intervention development and evaluation are conducted within the framework of the multi-center interdisciplinary COACH project. COACH focusses on AYA with cystic fibrosis (CF), juvenile idiopathic arthritis (JIA) or T1D. The reason for selecting these chronic medical conditions is to cover a broad spectrum of diseases with different requirements and burdens for AYA. In order to develop a user-centered iCBT program and thus to increase the effectiveness of and engagement with youthCOACH_CD,_ the intervention program is developed in three steps. In a first step, qualitative research is conducted to determine concerned AYA needs and preferences for the program [27]. Second, a feasibility trial is conducted to ensure the acceptance, feasibility and potential efficacy of youthCOACH_CD_. Third, a large scale randomized controlled trial is conducted to evaluate the (cost-) effectiveness of youthCOACH_CD_ [28]. Here we report on the results of the second step, the feasibility trial. As recommended by the CONSORT statement, the primary aim of these studies is the assessment of the feasibility of conducting a future definitive trial [29]. Therefore, we focus on the following objectives:

1. Is youthCOACH_CD_ feasible and accepted as measured by adherence and dropout rates, formative user feedback, potential side effects, interventions satisfaction, and therapeutic alliance?
2. Does youthCOACH_CD_ potentially have a short- and medium-term efficacy in terms of improved symptoms of anxiety and depression, coping with the disease, health-related quality of life, self-efficacy, post-traumatic growth, reduced symptoms of post-traumatic stress disorder, less alcohol consumption, and perceived social support in comparison to a waiting list control group?

## Methods

A randomized controlled feasibility trial was conducted comparing the guided iCBT youthCOACH_CD_ (IG) with a waiting list control group (CG) receiving the unguided youthCOACH_CD_ program six months post-randomization. The study is reported and was conducted in accordance with the CONSORT statement for feasibility trials [29], approved by the ethics committee of Ulm University (Number 292/18) and a-priori registered at the WHO International Clinical Trials Registry Platform via the German Clinical Trials Register (ID: DRKS00016714, 25/03/2019). Please note, we changed the primary study aim from potential efficacy (as described in the study registration) to feasibility in order to emphasize the explorative design of this trial.

### Participants

AYA (1) aged between 12 and 21 years, (2) reporting to have CF, JIA or T1D (self-report) were eligible for participation in case of (3) available internet access and (4) providing informed consent for participation. We included AYA with a broad range of symptoms of anxiety and depression in order to generate knowledge regarding the accessibility, usefulness and feasibility of youthCOACH_CD_ across the mental health strains spectrum. Therefore, there was no cut-off scores for the self-reported symptoms of depression and anxiety to be involved in the study. Additionally, having a score > 1 in the ninth item of the Beck Depression Inventory revision (BDI-II item = 0: “*I’m not thinking of harming myself.*”, BDI-II item = 1: “*I have thoughts of killing myself, but I would not carry them out*.”, BDI-II item = 2: “*I would like to kill myself.*”, BDI-II item = 3: “*I would kill myself if I had the chance.*”) at baseline measurement was an exclusion criterion [30]. Participants stating a BDI-II Item 9 = 1, received an online information letter with detailed information on available health services and the advice to use professional help, but were not excluded from the study. All inclusion criteria were based on the self-reports of the participants.

### Recruitment of Participants

Between April 2019 and May 2020 an open recruitment strategy was applied to AYA living in Germany: social media posts in chronic condition related self-help groups, flyers in medical practices and clinics, or information to e-mail distribution lists of self-help groups. Interested AYA contacted the study team via e-mail. AYA eligible for inclusion had to return their informed consent, in case of AYA younger than 16 years, informed consent had to be provided by caregivers as well. Additionally, participants were asked to name a caregiver for others assessments about the AYA. Caregivers needed to be 18 years of age or older and provide written informed consent for inclusion as third person assessor of AYAs’ health.

### Sample Size

A formal sample size calculation was not performed, given the feasibility character of this trial. The number of participants was estimated at around 10% of the calculated sample size required for the definitive RCT (*n* = 212) and we anticipated a high dropout rate of 50%, resulting in a sample size of *n* = 30 [31].

### Randomization

Randomization was performed at the individual level by a person not otherwise involved in the trial process applying a permuted block randomization with an allocation ratio of 1:1 and variable 4, 6 and 8-block sizes. Random allocation was computer-generated using the program Sealed Envelope [32]. Members of the study team who were concerned with outcome assessment, were blinded and did not receive any information about the participants’ group allocation.

### Intervention Protocol

The intervention program youthCOACH_CD_ was developed by the department of clinical psychology and psychotherapy at Ulm University. youthCOACH_CD_ is CBT-based and consists of an introductory session and seven modules. A description of the specific module contents can be found elsewhere [28]. All intervention contents are presented in a youth-oriented and varied manner (i.e. video-, audio-, illustration- and text-based content presentation; writing-based or multiple-choice tasks and quizzes). Stories of three fictional AYA with a chronic condition and symptoms of depression and anxiety are implemented in every module in order to facilitate identification. Intervention content and design were informed by focus groups conducted with potential users of youthCOACH_CD_ [27].

AYA were recommended to complete one module per week. AYA in the IG were allocated to one of two eCoaches who were graduates of a Master’s Degree in Psychology and were enrolled in a training program in CBT with children and adolescents. eCoaches were supervised by two licensed psychotherapists and provided semi-standardized, asynchronous feedback after each completed module, including positive reinforcement and motivation, based on principles of CBT [28]. Participants of the IG were asked to schedule an appointment for completing their next module and were reminded by their eCoach, if they did not stick to their appointment or managed to work on their modules for an extended period of time. First, three reminding messages of the eCoach were sent to the participant (2, 5 and 8 days after the scheduled appointment). In order to use an additional contact medium, the non-blinded members of the study team attempted to reach the participant by phone, three times over a period of two weeks. Participants were asked for potential reasons for not completing their modules (e.g. technical difficulties). If the contact attempt failed, these participants were regarded as dropouts.

In addition to the seven modules, AYA were advised to keep a daily mood diary, which is offered on a mobile app [33]. This was intended to support the transfer of the intervention contents into everyday life. Furthermore, the app provided daily motivational prompts.

youthCOACH_CD_ was implemented via the secure online platform Minddistrict [33]. Participants of the CG were given access to the unguided version of youthCOACH_CD_ six months after the randomization. Participants of both the CG and the IG could make use of any offers of additional routine care and were informed about these via flyer.

### Measurements

Participants of both the IG and the CG were invited to participate in self-report surveys at three time points: Baseline (t0), twelve weeks post-randomization (t1) and six months post-randomization (t2, follow-up measurement), regardless of the extent to which AYA of the IG have completed the intervention process. Surveys were implemented on the secure platform Unipark [34]. Participants were invited by e-mail. If they did not respond to the invitation, they were reminded by e-mail and phone calls. AYA received 10€ for each completed survey as compensation. Named caregivers also completed surveys at the same three measurement points. Table 1 presents an overview of all applied measurement instruments and assessment time points. A detailed description of the measurement instruments can be found elsewhere [28].

**Table 1:** Outcome assessment and assessment time point, if applicable

Variables	Measurement	Baseline	t1	t2
**Measures of Feasibility**				
Subjective side effects	Inventory for Recording Negative Effects of Online Interventions (INEP-On) [35]		X (IG only)	X (IG only)
Internet usage behavior	Internet-Use Expectancies Scale (IUES) [36]	X	X	X
Therapeutic alliance	Working Alliance Inventory-Short Revised (WAI-SR) [37, 38]		X (IG only)	X (IG only)
Client Satisfaction	Client Satisfaction Questionnaire adapted to Internet-based interventions (CSQ-I) [39]		X (IG only)	X (IG only)
Formative user feedback	Participants of the IG had the voluntary opportunity to provide formative feedback on: - the duration - positive and negative aspects of each module	*After each module* (IG only)		
Study dropout	Absolute numbers and percentages of participants not completing all measurement points			
Intervention adherence	Adherence in the IG was defined as completion of the introduction lesson and 80% of the seven modules up to t1			
Daily mood diary	Median usage of daily mood diary			
**Measures of efficacy**				
Depressive and anxiety symptom severity	Patient Health Questionnaire Anxiety and Depression Scale (PHQ-ADS) [40]	X	X	X
Health-related quality of life	EuroQol Five-Dimensional Questionnaire - Youth (EQ-5D-Y) [41, 42]	X	X	X
Coping	Coping with a Disease (CODI) [43]	X	X	X
Self-efficacy	General perceived Self-Efficacy scale (GSE) [44]	X	X	X
Symptoms of posttraumatic stress disorder	The Child and Adolescent Trauma Screen (CATS) 7-17 [45]	X	X	X
Personal growth	Short version of the Stress-Related Growth Scale (SRGS) adapted to AYA with chronic conditions [46, 47]	X	X	X
Social Support	Sub-scale “Actually received support, recipient” of the Berliner Social Support Scale (BSSS) [48]	X	X	X
Alcohol consumption	Three alcohol consumption questions from the Alcohol Use Disorders Identification Test (AUDIT-C) [49]	X	X	X
**Further measurements**				
Service utilization; medications taken; medical devices used	Child and Adolescents Service Receipt Inventory German Version (CAMSHRI-DE) [50]	X	X	X
Behavioral activation	Behavioral Activation for Depression Scale (BADS) [51]	X	X	X
Automatic thoughts	Automatic Thoughts Questionnaire-Revised (ATQR) [52]	X	X	X
**Caregiver reports**				
Symptoms of anxiety	Screen for Child Anxiety Related Emotional Disorders (SCARED) [53]	X	X	X
Symptoms of depression	Mood and Feeling Questionnaire (SMFQ) [54]	X	X	X
Symptoms of posttraumatic stress disorder	Child and Adolescents Trauma Screen-Caregiver (CATS-C-D) [45]	X	X	X
Social Support	Subscale “Actually received support, provider” of the Berliner Social Support Scale (BSSS) [48]	X	X	X

*Note.* t1 = 12 weeks post-randomization; t2 = 6 months post-randomization; IG = Intervention group. For detailed information on the psychometric properties of the measurements, please see [28]

### Statistical Analysis

To answer the main objective of this study – the feasibility of youthCOACH_CD_ – the following measurements were analyzed: adherence and dropout rates, formative user feedback, potential side effects, intervention satisfaction, and therapeutic alliance. Adherence and dropout rates, participant scores in the assessment of intervention satisfaction, as well as therapeutic alliance were compared to the results reported in other clinical trials on IMI targeting AYA. Comparable results pointing to feasibility of the trial were discussed. Furthermore, potential side effects were measured by the INEP-On as well as the Internet Usage Expectancies Scale. If a pattern in the participants’ answers on the INEP-On had emerged, that would have been discussed as a side effect. Additionally, decreased internet usage would have be rated as another side effect.

Data was analyzed using R [55]. Participants’ characteristics and all outcome variables are described descriptively (mean, standard deviation, frequency, percentage). Potential group differences at post-randomization measurement points are investigated using linear regression models, adjusting for baseline values for continuous outcomes. All continuous variables were z-standardized, and dummy-coding was applied to dichotomous variables. Potential outliers, which bear the risk of biasing results, were identified using scatter plot and Cook`s distance [56–58]. Because the sample size in this feasibility trial has low statistical power, corresponding 95%-confidence intervals are reported for each outcome, but we refrained from reporting p-values [29, 59]. Furthermore, the standardized regression coefficient as well as Cohen’s d for between group differences and the corresponding 95%-confidence intervals are reported. Sensitivity analysis showed no crucial differences between data imputed by multivariate imputation by chained equations [60] with predictive mean matching method and observed data. Therefore, we decided to report on the observed data only. All efficacy analyses are based on an intention-to-treat (ITT). Furthermore, to get a comprehensive idea of the potential effectiveness, analysis based on per-protocol (PP) principle were conducted. PP was defined as participants completing 80% of the modules up to t1. In the forthcoming confirmative RCT, only AYA with clinically relevant symptoms of depression and/ or anxiety will be involved. In this feasibility trial we also included AYA without clinically relevant symptoms due to ethical reasons. Therefore, additional sub-group analyses for AYA with clinically relevant symptoms of depression and / or anxiety (PHQ-9 or GAD-7 score ≧ 7) [61, 62] were conducted. Because of this feasibility trial’s low sample size, a cost-analysis based on the CAMSHRI-DE is not applicable. Therefore, we only conducted descriptive analysis of service use according to the CAMSHRI-DE.

Regarding the CONSORT guidelines for feasibility trials, the analysis of mediating and moderating effects with a sample of *n* = 30 is not applicable [29]. Therefore, only changes in behavioral activation and automatic thoughts that might have a mediating effect, are reported.

## Results

### Recruitment and Baseline Characteristics of Participants

Thirty-three AYA were assessed for eligibility and provided informed consent. Thirty AYA completed baseline measurements and were included for randomization. The mean age of randomized AYA was *M* = 16.13 (*SD* = 2.34). Of the participants, 73% were female. Furthermore, 13% of AYA reported having CF, 37% JIA, and 50% T1D. Average depression and anxiety symptom burden was *M* = 12.83 (*SD* = 7.48). Detailed information on baseline sample characteristics is provided in Table 2. Of participants (IG: *n* = 8; CG: *n* = 7), 50% showed symptoms of anxiety and/ or depression above a cut-off score of PHQ-9 or GAD-7 ≥ 7.

Almost all participants reported having been treated by a general practitioner or paediatrician (24/30) and a disease-specific physician (29/30) during the 9-month reporting period. Several participants reported having used further medical/therapeutic care services: inpatient care (8/30), physiotherapy (13/30), psychotherapy (6/30) and ergotherapy (5/30). School-based services (especially additional support through the class teacher) and social services were only used by a small percentage of the participants (6/30 or 5/30, respectively). The median number of medications taken or medical devices used is 2.0 each (supplementary material Table A.2).

**Table 2:** Participant characteristics at baseline per group and overall

	Intervention Group (*n* = 15)	Control Group (*n* = 15)	All Participants (*N* = 30)
Gender, %	80% female	66% female	73% female
Age, M (SD)	16.06 (2.37)	16.20 (2.40)	16.13 (2.34)
Medical condition, n (%) CF JIA T1D	3 (20%) 5 (33%) 7 (47%)	1 (7%) 6 (40%) 8 (53%)	4 (13%) 11 (37%) 15 (50%)
Relationship, %	80% single	80% single	80% single
Education, n Comprehensive School ^a^ High School^b^ Grammar School Studies ongoing Job Job Training Other	0 2 6 3 0 1 3	1 1 7 1 1 1 3	1 3 13 4 1 2 6

*Note.* ^a^ German “Haupt-/Volksschule”; ^b^ German , Realschule“

### Feasibility Outcomes

#### Intervention adherence

Of the participants in the IG (*n* = 15), nine (60%) completed all seven modules; one completed three modules (7%); one completed the introductory module (7%) and four (27%) never began the intervention. Reasons for no uptake of the intervention were: no motivation, changes in life circumstances, too much time or effort required. AYA who completed all seven modules needed between five to 32 weeks. The participant who needed 32 weeks interrupted the intervention for personal reasons. The median for intervention duration was eleven weeks. 33% of AYA completing all modules completed questionnaires twelve weeks post-randomization before they had finished all modules.

Of the IG, six participants completed 80% of the modules up to t1 (a-priori defined as adherent; 40%). This subsample was included in per protocol analyses on efficacy outcomes.

AYA of the IG used the daily mood diary 0 to 46 times, with a median of 6 times. Including only adherent participants of the IG, median usage was 19 times (range: 1- 46).

#### Study dropout

**Fig. 1:**
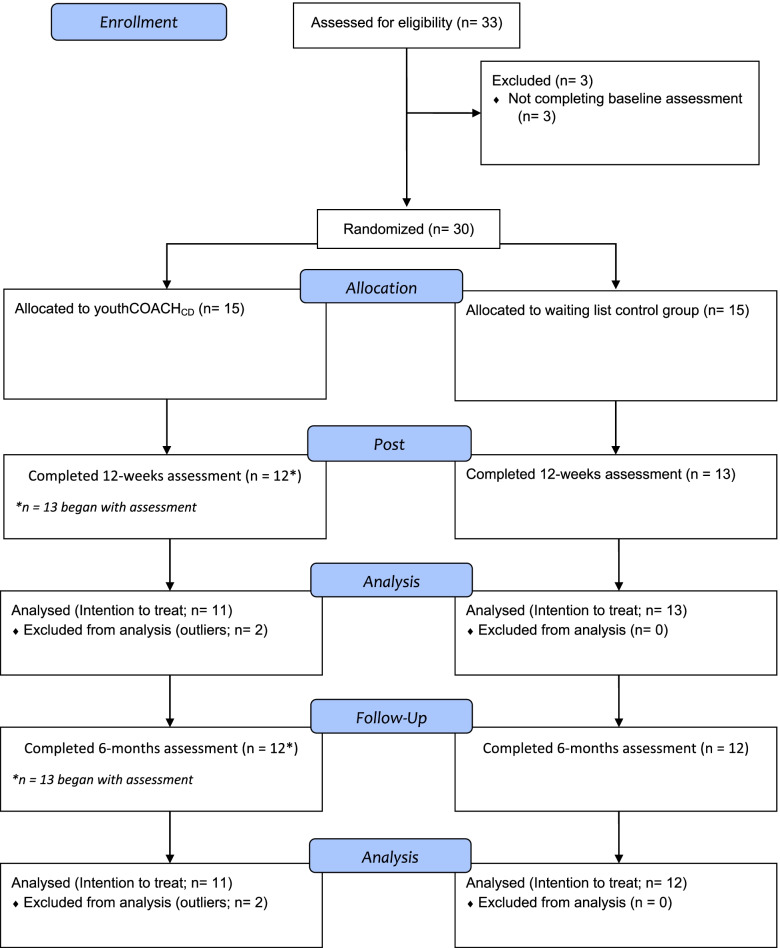
CONSORT flow chart displaying participant flow through the study

Of the included 30 participants, 24 completed all questionnaires (80%; IG: 80%, CG%: 80%), pointing to a study dropout of 20% (Fig. 1).

#### Formative feedback

Seven participants of the IG provided feedbacks across the seven modules on time needed per module: 33% reported to spent 10-30 min, 54% 30-50 min, 7% 50-70 min, 3% 70-90 min and 3% 90-120 min. 87% perceived the length of the modules as appropriate, 5% as too long and 8% as too short. No AYA provided negative qualitative feedback. 17 positive qualitative feedbacks across modules were provided. These solely included individual tasks that were perceived to be particularly helpful.

#### Subjective side effects

Potential side effects of the participation in youthCOACH_CD_ were assessed twelve weeks and six months post-randomization in the IG. Based on INEP-On, AYA of the IG reported overall 45 negative, 146 neutral, and 66 positive effects twelve weeks after randomization and 41 negative, 149 neutral, and 66 positive effects six months post-randomization. Eight AYA reported at least one negative effect attributed to the intervention. In detail, at twelve weeks and six months post-randomization AYA linked 22% (*n* = 10) and 32% (*n* = 13) of the negative effects, 24% (*n* = 35) and 24% (*n* = 36) of the neutral effects, and 67% (*n* = 44) and 73% (*n* = 48) of the positive effects to the intervention. The most often reported negative effects twelve weeks post-randomization were difficulties in making decisions alone (*n* = 5, 0% attributed to intervention), longer periods of feeling bad (*n* = 5, 0% attributed to intervention) and decreased motivation to start a psychotherapeutic treatment (*n* = 5, 80% attributed to intervention). The most reported negative effects six months post-randomization were: financial worries (*n* = 5, 20% attributed to intervention) and difficulties in making decisions alone (*n* = 5, 0% attributed to intervention). A detailed overview for both measurement time points can be found in the supplementary material (Table A.3 and Table A.4).

#### Suicidal ideation

Suicidal thoughts without intention to realize them, occurred twice at baseline measurement, once at t1, and twice at t2. The participants stated a BDI-II Item 9 = 1.

#### Satisfaction with intervention

Of the IG, twelve AYA reported mean satisfaction with youthCOACH_CD_ of *M* = 25.42 (*SD* = 5.85) at twelve weeks post-randomization and of *M* = 26.50 (*SD* = 4.68) six months post-randomization (measured by the CSQ-I: potential sum score range 8-32). In addition, 58% would recommend the intervention to a friend, 17% would likely recommend it, 17% would partly recommend it, and 8% would not recommend youthCOACH_CD_ to a friend.

#### Therapeutic alliance

In the IG, twelve participants rated the therapeutic alliance with the WAI-SR questionnaire (range per scale: 1-5) twelve weeks post-randomization on the scales bond (*M* = 2.77, *SD* = 1.26), task (*M* = 2.82, *SD* = 1.20) and goal (*M* = 2.91, *SD* = 1.35). The overall mean was *M* = 2.83 (*SD* = 1.25). The six months post-randomization bond was rated with *M* = 2.75 (*SD* = 1.13), task with *M* = 3.06 (*SD* = 1.06) and goal with *M* = 3.15 (*SD* = 0.97). Resulting in an overall mean of 2.99 (*SD* = 1.00) six months post-randomization.

#### Internet usage

The internet usage behavior (in hours per week) decreased in the IG (see Table 3). Additionally, changes in expectations of using the internet measured by the IUES are presented in Table 4.

### Efficacy outcomes

Graphical illustration and Cook’s distance revealed two outliers regarding symptom change (PHQ-ADS) from baseline to twelve weeks post-randomization. Both outliers belonged to the IG. In ITT-analysis, there were non-significant changes in the primary efficacy outcome, symptoms of anxiety and depression, in the IG in comparison to the CG twelve weeks post-randomization (β = 0.20, 95%-CI: [-0.47; 0.88]) and six months post-randomization (β= -0.55, 95%-CI: [-1.17; 0.07]) when adjusting for baseline values. Table 3 presents all means and standard deviations for all outcomes in the CG and IG. Data on all efficacy outcomes are presented in Table 4.

Subsample analysis based on ITT-analysis, including *n* = 14 participants reporting symptoms of anxiety and/ or depression above a cut off score of PHQ-9 or GAD-7 ≥ 7 at baseline measurement (IG: *M* = 21.41, *SD* = 16.71; CG: *M* = 16.71, *SD* = 4.53) revealed non-significantly higher symptom scores (PHQ-ADS) in the IG compared to the CG twelve weeks post-randomization (β = 0.27, 95%-CI: [-0.96; 1.51]) and non-significantly lower symptom scores (PHQ-ADS) six months post-randomization (β = -0.53, 95%-CI: [-1.84; 0.77]), adjusted for baseline scores. Data on all efficacy outcomes, based on participants with clinically relevant or no clinically relevant symptoms of anxiety and depression, are presented in the supplementary material (Table A.5 and Table A.6).

Additionally, per protocol analysis showed non-significant difference between the IG and the CG regarding PHQ-ADS score twelve weeks post-randomization (β = 0.07, 95%-CI: [-0.78; 0.91]) and six months post-randomization (β = -0.52, 95%-CI: [-1.33; 0.28]), adjusted for baseline scores. Detailed information on results of the per protocol analysis can be found in the supplementary material (Table A.7).

**Table 3:** Means and standard deviations for the intervention group and waiting list control group

	Baseline (t0)				12 weeks post-randomization (t1)				6 months post-randomization (t2)			
	IG (*n* = 13)		CG (*n* = 15)		IG (*n* =11)		CG (*n* =13)		IG (*n* =11)		CG (*n* =12)	
**Outcome**	*M*	*SD*	*M*	*SD*	*M*	*SD*	*M*	*SD*	*M*	*SD*	*M*	*SD*
PHQ-ADS	14.77	8.96	11.40	6.31	11.18	7.81	9.38	4.66	10.91	9.74	13.83	7.20
VAS*	70.31	17.20	73.00	18.90	75.09	20.00	69.54	17.96	69.20	24.75	63.75	25.37
CODI*	77.92	8.30	80.80	8.76	76.00	6.03	81.08	8.62	77.80	7.60	80.83	9.34
GSE*	24.69	5.30	29.07	5.50	28.73	4.86	30.15	4.49	27.80	4.76	29.33	4.62
CATS	17.08	9.12	12.87	7.52	13.18	9.80	14.85	7.99	11.50	11.99	15.75	7.64
SRGS*	16.54	7.24	14.00	6.62	16.73	7.18	15.08	7.18	17.80	9.54	12.17	5.52
AUDIT	1.92	2.50	2.27	2.15	1.82	2.23	1.46	1.56	1.50	1.90	1.75	1.82
BSSS*	43.38	4.57	41.60	4.45	43.00	6.45	42.08	4.03	42.70	5.93	43.91	6.82
IUES	27.31	9.11	26.33	7.00	24.45	7.30	24.08	8.83	24.50	9.79	26.00	8.06
Usage-I	20.84	20.87	15.38	8.58	16.45	13.06	15.54	18.19	6.36	28.97	14.67	12.07
Usage-S	12.85	25.93	17.92	15.30	18.82	10.64	17.96	24.85	5.18	24.35	15.92	13.64
BADS*	77.23	18.60	90.13	14.01	83.91	18.02	92.69	16.47	88.00	16.64	86.00	18.81
ATQ-R	33.69	17.38	28.33	13.80	28.36	15.34	23.30	12.79	31.20	20.48	29.75	15.26

*Note.* IG = Intervention group; CG = Control group; M = Mean; SD = Standard deviation; PHQ-ADS = Patient Health Questionnaire Anxiety and Depression Scale [score range: 0-48]; VAS = Visual Analogue Scale of the EuroQol Five-Dimensional Questionnaire- Youth [score range: 0-100; detailed analysis of EuroQol subscales can be found in Table A.1]; CODI = Coping with a Disease [score range: 28-140]; GSE = General perceived Self-Efficacy scale [score range: 10-40]; CATS = Child and Adolescent Trauma Screen [score range: 0-60]; SRGS = Stress-Related Growth Scale [score range: 0-30]; AUDIT-C = Alcohol Use Disorders Identification Test [consumption items; score range: 0-12]; BSSS = Berliner Social Support Scale [subscale: Actually received support, recipient; score range 12-48]; IUES = Internet Use Expectancies Scale [score range 8-48]; Usage-I = Internet usage in hours per week; Usage-S = Smartphone usage in hours per week; BADS = Behavioral Activation for Depression Scale [score range: 0-125]; ATQ-R= Automatic Thoughts Questionnaire-Revised;

* higher scores indicate better outcome

**Table 4:** Results of linear regression models and Cohen’s d for efficacy outcomes at post-treatment (t1) and 6-month follow-up (t2) based on ITT analyses

	12 weeks post-randomization (t1)		6 months post-randomization (t2)	
	Standardized regression coefficient (95% CI)^a^	Between-group effect size Cohens d (95% CI)	Standardized regression coefficient (95% CI)^a^	Between-group effect size Cohens d (95% CI)
**Outcome**				
PHQ-ADS	0.20 [-0.47; 0.88]	0.30 [-0.52; 1.10]	-0.55 [-1.17; 0.07]	-0.36 [-1.18; 0.48]
CODI*	-0.18 [-0.83; 0.48}	-0.70 [-1.54; 0.16]	0.15 [-0.44; 0.74]	-0.37 [-1.21; 0.49]
VAS*	0.24 [-0.52; 1.00]	0.31 [-0.51; 1.11]	0.22 [-0.63; 1.07]	0.23 [-0.62; 1.07]
GSE*	0.11 [-0.39; 0.61]	-0.32 [-1.13; 0.50]	0.15 [-0.55; 0.85]	-0.34 [-1.19; 0.52]
SRGS*	-0.19 [-0.74; 0.37]	0.24 [-0.57; 1.04]	0.29 [-0.33; 0.90]	0.78 [-0.14; 1.66]
CATS	-0.36 [-0.97; 0.26]	-0.20 ([-1.00; 0.61]	-0.67 [-1.26; -0.08]	-0.45 [-1.3; 0.42]
AUDIT-C	0.09 [-0.30; 0.48]	0.20 [-0.61; 1.00]	0.04 [-0.64; 0.71]	-0.14 [-0.98; 0.70]
BSSS*	0.04 [-0.69; 0.69]	0.18 [-0.63; 0.99]	-0.29 [-1.13; 0.56]	-0.20 [-1.04; 0.65]
IUES	0.10 [-0.58; 0.78]	0.05 [-0.76; 0.85]	-0.22 [-0.77; 0.33]	-0.18 [-1.02; 0.67]
BADS*	-0.10 [-0.77; 0.57]	-0.53 [-1.35; 0.31]	0.64 [-0.02; 1.30]	0.12 [-0.73; 0.96]
ATQ-R	0.39 [-0.21; 0.99]	0.38 [-0.45; 1.19]	0.06 [-0.54; 0.70]	0.09 [-0.76; 0.92]

*Note.* CI= Confidence Interval; PHQ-ADS = Patient Health Questionnaire Anxiety and Depression Scale; CODI = Coping with a Disease; VAS = Visual Analogue Scale of the EuroQol Five-Dimensional Questionnaire- Youth; GSE = General perceived Self-Efficacy scale; SRGS = Stress-Related Growth Scale; CATS = Child and Adolescent Trauma Screen; AUDIT-C = Alcohol Use Disorders Identification Test (consumption items); BSSS = Berliner Social Support Scale (subscale: Actually received support, recipient); IUES = Internet Use Expectancies Scale; BADS = Behavioral Activation for Depression Scale; ATQ-R= Automatic Thoughts Questionnaire-Revised

* higher scores indicate better outcome; ^a^ controlling for baseline scores

### Caregiver report

Baseline measurements were provided by twenty caregivers (IG: *n* = 10; CG: *n* = 10). Of these 19 (IG: *n* = 10; CG: *n* = 9) caregivers also provided measurements twelve weeks or six months post-randomization (95% female, *M*_age_ = 45.51, *SD*_age_ = 5.15). Symptoms of anxiety (β = -0.30, 95%-CI: [-1.16; 0.57]) or depression (β = -0.18, 95%-CI: [-0.93; 0.58]) of AYA reported by caregivers have not significantly changed twelve weeks and six months (anxiety: β = -0.20, 95%-CI: [-1.14; 0.73]; depression: β = - 0.20, 95%-CI: [-0.84; 0.44]) post-randomization. Detailed information on mean and standard deviation on all caregiver reported outcomes as well as results of linear regression analyses of all other caregiver reported outcome changes can be found in the supplementary material (Table A.8 and Table A.9).

## Discussion

This randomized controlled pilot trial evaluated the feasibility and potential efficacy of an iCBT for AYA with a chronic medical condition and comorbid symptoms of anxiety and/ or depression. To our knowledge, this is the first iCBT intervention for this specific target group in Germany. Feasibility of youthCOACH_CD_ was supported and no severe side effects were reported. Feasibility was operationalized by adherence and dropout rate, formative user feedback, potential side effects, interventions satisfaction, and therapeutic alliance.

We observed a study dropout rate of 20%. In comparison to reported treatment dropout rates from efficacy studies among children and adolescents in an outpatient mental health care setting [63] as well as to these reported from internet-based interventions for mental disorders in AYA [64, 65], this rate is above average. Hence, the applied close reminder procedure by e-mail and phone should be maintained.

The observed intervention adherence rate of 40% in the IG is restricted but comparable to common adherence rates reported in self-management IMIs targeting AYA [66]. It might be valuable to consider further strategies to increase intervention adherence. In addition to the aforementioned close reminder procedure by the study team or the eCoaches, strategies of the Persuasive System Design principle (PSD; [67]) could be valuable in order to prevent non-adherence during intervention completion. Principles of the PSD framework aim to optimize the human-machine interaction and therefore influence users’ attitudes and behaviors [68]. Implementing PSD in IMI showed positive effects on treatment adherence, effectiveness and user satisfaction before [66, 69]. Increasing the trustworthiness and competence of an intervention is one of the four PSD key principles (system credibility). Given that twelve weeks post-randomization 33% of participants reported concerns about data security (INEP-On), optimizing youthCOACH_CD_ with regard to system credibility could be valuable. Additionally, improving the interaction between the user and intervention system is a further core feature of the PSD [67]. One potential strategy to realize this principle could be the implementation of further gamification approaches [70]. In the current version of youthCOACH_CD_, participants have the opportunity to download a certificate for every completed module. This reward procedure might be more effective if rewards would be presented in a more gamified manner, e.g. by collecting badges or trophies for every completed task [71, 72]. Furthermore, an avatar accompanying the AYA through the intervention could increase the social role and liking of the intervention and therefore might be valuable for users’ adherence [73]. Incorporating peer support is a further PSD principle. For example, consideration could be given to including a feature that allows participants to remind each other to complete the mood diary and thus collect rewards together. This could potentially increase the reported low engagement with the mood diary. However, further studies are required regarding the potential effectivity of such peer support features [69]. Participants of the focus groups which were conducted before the development of youthCOACH_CD_ did not reach consent whether they would like such peer support features [27]. As an interesting additional finding, the unguided version of youthCOACH_CD_ as provided to the wait list control group resulted in an intervention uptake rate of only 53% (uptake rate in IG: 73%) and a completer rate of 7% (completer rate in IG: 60%). Hence, human guidance seems to be a decisive factor regarding intervention uptake and adherence. This result is consistent with the literature indicating that guided IMI are superior to unguided versions [74, 75]. In the forthcoming RCT, the involvement of recruiting physicians could be valuable to further strengthen AYAs’ adherence [79]. Physicians might reinforce intervention uptake by using strategies such as motivational interviewing [76]. Furthermore, they could continually ask the AYA about completing the modules to reinforce a certain commitment of AYA. Another important aspect in regard to adherence might be parental involvement [77, 78]. In our focus groups study, AYA pointed out that they would prefer not to involve their parents [27]. However, youthCOACH_CD_ targets a wide age range of AYA. Younger AYA might be supported by parental involvement in a different way than older AYA. The potential moderating effect of age on adherence and effectiveness will be part of the forthcoming confirmative RCT. The potential benefits of parental involvement should be assessed in future dismantling studies.

Intervention content was based on established methods of CBT and informed by focus groups preliminary to this study [27]. Intervention content should be perceived as personal relevant and supportive to increase intervention adherence and effectiveness of an IMI [79]. Formative user feedback showed that AYA completing intervention modules had no negative feedback about intervention content. Therefore, the intervention content seems to be perceived as appropriate for this target group. Furthermore, with 87% rating the length of modules to be adequate, no further modifications might be required.

Further results on the feasibility of youthCOACH_CD_ are promising. The therapeutic alliance is comparable to those in other internet-based interventions [38, 80]. No pattern emerged regarding reported negative subjective side effects of the intervention attributed to youthCOACH_CD_ and neither the internet usage nor the internet use expectancies changed importantly in the IG. However, the high standard deviations limit the interpretability of the results. Nevertheless, it is important to emphasize at this stage that there are no indications of a subjective and objective negative side effect due to participation in youthCOACH_CD_.

More female (73%) than male AYA participated. One possible explanation could be that females are more affected by internalizing disorders during adolescence [9]. Furthermore, more females are affected by JIA [81]. In addition, male adolescents with emotional burdens tend to show less help-seeking behavior than female adolescents [82, 83]. Some of the known barriers to help-seeking in AYA are self-reliance, lack of perceived confidentiality and trust in the potential source of help, or lack of knowledge about symptoms of mental disorders [84]. In order to increase intervention reach, these potential barriers should be addressed in the recruitment process, e.g., through personal information provided by a trusted physician or applying strategies of motivational interviewing [76, 85, 86].

Results on potential efficacy of youthCOACH_CD_ should be interpreted with caution and in an explorative manner due to the small underpowered sample size and the overall explorative nature of this feasibility trial [59, 87]. The effect was not yet found twelve weeks post-randomization. The explorative interpretation of the study findings might suggest a temporary worsening of symptom. It is common that especially treatment of anxiety or post-traumatic stress disorder in AYA bears the risk of no improvement or symptom increase during or short-term after treatment [88]. This might be a necessary feature of effective psychotherapy [89]. However, follow-up measurements often reveal a positive long-term effect on symptom change as it is the case in this feasibility trial. The assumption of initially symptom worsening by confrontation with the mental disorders in this study, might be supported by the changes in potential mechanisms of change: e.g. automatic negative thoughts, measured by the ATQ-R, increased twelve weeks post-randomization, meaning, that maladaptive thought patterns seem to be more present short term after intervention and decrease to follow-up. Similar patterns can be seen in further potential mechanisms of change, i.e. coping with the disease and behavioral activation [90]. However, no final conclusion can be drawn until the results of the confirmatory trial.

Some limitations should be considered when interpreting the presented findings. The sample size is very small and no final conclusions on efficacy of youthCOACH_CD_ can be drawn. Furthermore, due to the small sample size, we only report on the efficacy of youthCOACH_CD_ across all three included chronic medical conditions. Efficacy of the iCBT and possible disease specific differences regarding efficacy could only be assessed in a larger sample or in disease specific studies. Stratified randomization is recommended in future similar trials to avoid unequal distribution of chronic medical conditions between study arms (e.g. 75% (3/4) of AYA with CF were in the IG in this study).

## Conclusions

Due to the challenging nature of living with a chronic medical condition in adolescence, it is important to provide low-threshold psychological support to this currently underserved target group. The adherence and dropout rates, intervention satisfaction, and therapeutic alliance results found in this randomized controlled pilot trial are comparable to the results of other internet-based trials. Formative user feedback was positive and no patterns emerged relating to any potential side effects of the intervention. These results point to the feasibility of youthCOACH_CD_, an iCBT for AYA with chronic medical conditions targeting symptoms of depression and anxiety. The results suggest that the implementation of a definitive future RCT is indicated to evaluate the effectiveness of the intervention and identify possible moderating and mediating effects.

## Supplementary Information

**Additional file 1.**

## Data Availability

The datasets used and/or analyzed during the current study are available from the corresponding author on reasonable request.

## Abbreviations

ATQ-R: Automatic Thoughts Questionnaire-Revised
AUDIT-C: Alcohol Use Disorders Identification Test (consumption items)
AYA: Adolescents and young adults
BADS: Behavioral Activation for Depression Scale
BSSS: Berliner Social Support Scale
CATS: Child and Adolescent Trauma Screen
CATS-C-D: Child and Adolescents Trauma Screen-Caregiver
CBT: Cognitive and Behavioral Therapy
CF: Cystic fibrosis
CG: Control group
CI: Confidence Interval
COACH: Chronic conditions in adolescents: implementation and evaluation of patient-centered collaborative healthcare
CODI: Coping with a Disease
Cohen’s d: Between-group effect size
GSE: General perceived Self-Efficacy scale
JIA: Juvenile idiopathic arthritis
iCBT: Internet-based cognitive behavioral therapy
IG: Intervention group
IMI: Internet- and mobile-based intervention
ITT: Intention to treat
IUES: Internet Use Expectancies Scale
M: Mean
PP: Per Protocol
PHQ-ADS: Patient Health Questionnaire Anxiety and Depression Scale
PSD: Persuasive System Design
SCARED: Screen for Child Anxiety Related Emotional Disorders
SD: Standard deviation
SMFQ: Short version of the Mood and Feeling Questionnaire
SRGS: Stress-Related Growth Scale
T1D: Type 1 Diabetes
VAS: Visual Analogue Scale of the EuroQol Five-Dimensional Questionnaire- Youth
β: Standardized regression coefficient
