# Supplementary material for: Feasibility and potential efficacy of a guided internet- and mobile-based CBT for adolescents and young adults with chronic medical conditions and comorbid depression or anxiety symptoms (youthCOACHCD): a randomized controlled pilot trial
Source: BMC Pediatr. 2022 Jan 29;22:69. doi: 10.1186/s12887-022-03134-3 (PMC8800235; doi:10.1186/s12887-022-03134-3)
Supplement: Supplementary file 1 — Additional file 1. [file 12887_2022_3134_MOESM1_ESM.docx]

### **Supplementary material**

**Table A.1** Detailed analysis of life quality by EQ-5D-Y

|  | Baseline (t0) | | 12 weeks post-randomization (t1) | | 6 months post-randomization (t2) | |
| --- | --- | --- | --- | --- | --- | --- |
|  | IG  (*n* = 15) | CG  (*n* = 15) | IG  (*n* =12) | CG  (*n* = 13) | IG  (*n* = 12) | CG  (*n* = 12) |
| Mobility (walking about) | |  |  |  |  |  |
| Some/ a lot of | 5 (66%) | 2 (13%) | 2 (17%) | 2 (15%) | 2 (17%) | 3 (25%) |
| Looking after myself |  |  |  |  |  |  |
| Some/ a lot of | 2 (13%) | 0 (0%) | 1 (8%) | 0 (0%) | 1 (8%) | 1 (8%) |
| Doing usual activities |  |  |  |  |  |  |
| Some/ a lot of | 6 (40%) | 3 (20%) | 2 (17%) | 2 (15%) | 2 (17%) | 2 (17%) |
| Having pain or discomfort | |  |  |  |  |  |
| Some/ a lot of | 5 (33%) | 6 (40%) | 3 (25%) | 5 (38%) | 4 (34%) | 7 (58%) |
| Feeling worried, sad, or unhappy | |  |  |  |  |  |
| Some/a lot of | 5 (33%) | 5 (33%) | 3 (25%) | 7 (54%) | 5 (42%) | 5 (42%) |
| Current health state compared to one year ago | | |  |  |  |  |
| similar or worse | 10 (66%) | 9 60%) | 8 (67%) | 9 (69%) | 7 (58%) | 12 (100%) |

*Note.* IG = intervention group; CG = control group

**Table A.2** Detailed information on service use (CAMSHRI, frequency of users or Median) over a 9-month reporting period (3 months prior to t0 to t2)

|  | total | IG | CG | CF | JIA | T1D |
| --- | --- | --- | --- | --- | --- | --- |
| **Inpatient stay** | 8 | 2 | 6 | 2 | 5 | 1 |
| General hospital | 3 | 1 | 2 | 1 | 1 | 1 |
| Paediatric clinic | 4 | 1 | 3 | 1 | 3 | 0 |
| Psychiatric clinic | 0 | 0 | 0 | 0 | 0 | 0 |
| Rehabilitatation clinic | 1 | 0 | 1 | 0 | 1 | 0 |
| **Outpatient clinic** | 0 | 0 | 0 | 0 | 0 | 0 |
| **Outpatient physicians and therapists** | 30 | 15 | 15 | 4 | 11 | 15 |
| General physician | 14 | 7 | 7 | 3 | 5 | 6 |
| Paediatrician | 11 | 8 | 3 | 2 | 5 | 4 |
| Diabetologist | 14 | 7 | 7 | 0 | 0 | 14 |
| Otorhinolaryngologist | 6 | 4 | 2 | 4 | 0 | 2 |
| Rheumatologist | 11 | 5 | 6 | 0 | 11 | 0 |
| Psychotherapist | 6 | 3 | 3 | 0 | 6 | 0 |
| Ergotherapist | 5 | 3 | 2 | 0 | 5 | 0 |
| Physiotherapist | 13 | 8 | 5 | 4 | 8 | 1 |
| **School-based services^1^** | 6 | 1 | 5 | 2 | 4 | 0 |
| **Social services^2^** | 5 | 3 | 2 | 2 | 3 | 0 |
| **Medication and medical devices** |  |  |  |  |  |  |
| Number of medications | M = 2.0 | M = 3.7 | M = 1.0 | M = 11.5 | M = 2.0 | M = 1.0 |
| Number of medical devices | M = 2.0 | M = 2.0 | M = 2.0 | M = 2.5 | M = 0.0 | M = 2.0 |

*Note.* CF = Cystic fibrosis; JIA = Juvenile idiopathic arthritis; T1D = Type 1 diabetes; M = Median; IG = Intervention group; CG = Control group
^1^ e. g. class teacher, social worker, school companion; ^2^ e. g. children and youth emergency
service, Groups and activities for adolescent patients, online consulting

**Table A.3** Detailed information on reported subjective negative side effects (INEP-ON) 12 weeks post-randomization

|  | **Negative change** | | | **No change** | | | | **Positive change** | | | |
| --- | --- | --- | --- | --- | --- | --- | --- | --- | --- | --- | --- |
|  | n | By intervention | | n | | By intervention | | n | | By intervention | |
| **Intrapersonal change** |  |  | |  | |  | |  | |  | |
| Improvement/worsening of symptoms | 1 | 1 | | 3 | | 0 | | 8 | | 5 | |
| More/less trusting others | 1 | 0 | | 7 | | 1 | | 4 | | 4 | |
| Severity of suffering from past experiences/events | 0 | 0 | | 6 | | 2 | | 6 | | 4 | |
| Experiencing new thinking and behavior patterns as helpful/harmful | 0 | 0 | | 3 | | 1 | | 9 | | 8 | |
| More/ less feeling of loneliness | 0 | 0 | | 5 | | 1 | | 7 | | 3 | |
| Increased alcohol/ drug consume | 1 | 0 | | 11 | | 3 | | - | | - | |
| Feeling dependent on IMI program | 4 | 3 | | 8 | | 4 | | - | | - | |
| Difficulties to make decisions alone | 5 | 0 | | 7 | | 1 | | - | | - | |
| Longer periods of feeling bad | 5 | 0 | | 7 | | 3 | | - | | - | |
| As a human being changed to the negative | 2 | 1 | | 9 | | 3 | | - | | - | |
| Motivation to start psychotherapeutic treatment | 5 | 4 | | 4 | | 2 | | 3 | | 2 | |
| **Relationship, Friends, Family** |  |  | |  | |  | |  | |  | |
| More/less arguments/conflicts in relationship | 1 | 0 | | 2 | | 0 | | 3 | | 1 | |
| Problems in relationship/ family | 3 | 0 | | 9 | | 1 | | - | | - | |
| Worsened/improved relationship with family | 0 | 0 | | 6 | | 2 | | 6 | | 4 | |
| Worsened/improved relationship with friends | 0 | 0 | | 5 | | 1 | | 7 | | 3 | |
| **Stigma** |  |  | |  | |  | |  | |  | |
| Fear of others learning about the program usage | 1 | 0 | | 11 | | 4 | | - | | - | |
| Worries about (potentially) increasing insurance fees | 3 | 0 | | 9 | | 3 | | - | | - | |
| Financial worries | 4 | 0 | | 8 | | 3 | | - | | - | |
| **Therapeutic malpractice by online-training /eCoach** | | |  | |  | |  | |  | |  |
| Hurtful statements in online-training/by eCoach | 1 | - | | 11 | | - | | - | | - | |
| Feeling that the eCoach is supportive/ disruptive | 3 | 1 | | 2 | | 0 | | 7 | | 7 | |
| Feeling of data security not being ensured during the online-training | 4 | - | | 8 | | - | | - | | - | |
| Neglect of hobbies and social contacts because of online-training | 1 | 0 | | 5 | | 0 | | 6 | | 3 | |

*Note.* Data was observed in IG (n = 12) and reports on a 22-item version of the Inventory for the Assessment of Negative Effects of Psychotherapy developed for internet-based interventions (INEP-On).

**Table A.4** Detailed information on reported subjective negative side effects (INEP-ON) 6 months post-randomization

|  | **Negative change** | | **No change** | | | | **Positive change** | | | |
| --- | --- | --- | --- | --- | --- | --- | --- | --- | --- | --- |
|  | n | By intervention | n | | By intervention | | n | | By intervention | |
| **Intrapersonal change** |  |  |  | |  | |  | |  | |
| Improvement/worsening of symptoms | 1 | 1 | 2 | | 1 | | 9 | | 6 | |
| More/less trusting others | 0 | 0 | 4 | | 0 | | 8 | | 4 | |
| Severity of suffering from past experiences/events | 3 | 1 | 5 | | 1 | | 4 | | 3 | |
| Experiencing new thinking and behavior patterns as helpful/harmful | 1 | 0 | 3 | | 1 | | 8 | | 8 | |
| Feeling more/less lonely | 2 | 1 | 5 | | 0 | | 5 | | 4 | |
| Increased drug/ alcohol consume | 0 | 0 | 12 | | 2 | |  | |  | |
| Feeling dependent on IMI program | 1 | 1 | 11 | | 6 | | - | | - | |
| Difficulties to make decisions alone | 5 | 0 | 7 | | 1 | | - | | - | |
| Longer periods of feeling bad | 4 | 1 | 8 | | 3 | | - | | - | |
| As a human being changed to the negative | 3 | 2 | 9 | | 3 | | - | | - | |
| Motivation to start psychotherapeutic treatment | 2 | 2 | 6 | | 4 | | 4 | | 3 | |
| **Relationship, Friends, Family** |  |  |  | |  | |  | |  | |
| More/less arguments/conflicts in relationship | 0 | 0 | 4 | | 1 | | 0 | | 0 | |
| Problems in relationship/ family | 4 | 0 | 8 | | 1 | | - | | - | |
| Worsened/improved relationship with family | 0 | 0 | 7 | | 1 | | 5 | | 2 | |
| Worsened/improved relationship with friends | 1 | 0 | 4 | | 0 | | 7 | | 5 | |
| **Stigma** |  |  |  | |  | |  | |  | |
| Fear of others learning about the program usage | 2 | 2 | 10 | | 6 | | - | | - | |
| Worries about (potentially) increasing insurance fees | 4 | 0 | 8 | | 2 | | - | | - | |
| Financial worries | 5 | 1 | 7 | | 0 | | - | | - | |
| **Therapeutic malpractice by online-training /eCoach** | | | |  | |  | |  | |  |
| Hurtful statements in online-training/by eCoach | 0 | - | 12 | | - | | - | | - | |
| Feeling that the eCoach is supportive/ disruptive | 1 | 1 | 1 | | 1 | | 10 | | 9 | |
| Feeling of data security not being ensured during the online-training | 2 | - | 10 | | - | | - | | - | |
| Neglect of hobbies and social contacts because of online-training | 0 | 0 | 6 | | 2 | | 6 | | 4 | |

*Note.* Data was observed in IG (n = 12) and reports on a 22-item version of the Inventory for the Assessment of Negative Effects of Psychotherapy developed for internet-based interventions (INEP-On).

|  | 12 weeks post-randomization (t1) | | | | 6 months post-randomization (t2) | |  |
| --- | --- | --- | --- | --- | --- | --- | --- |
|  | | Standardized regression coefficient  (95% CI)^a^ | Between-group effect size Cohens d (95% CI) | Standardized regression coefficient  (95% CI)^a^ | | Between-group effect size Cohens d (95% CI) | |
| **Outcome** | |  |  |  | |  | |
| PHQ-ADS | | 0.27 [-0.96; 1.51] | 0.76 [-0.49; 1.95] | -0.53 [-1.84; 0.77] | | -0.01 [-1.10; 1.08] | |
| CODI* | | -0.09 [-1.30; 1.11] | -0.57 [-1.73; 0.64] | 0.14 [-0.44; 0.74] | | -0.27 [-1.36; 0.85] | |
| VAS* | | -0.31 [-1.61; 0.99] | -0.03 [-1.18; 1.11] | -0.26 [-1.37; 0.84] | | -0.22 [-1.31; 0.88] | |
| GSE* | | -0.23 [-1.13; 0.68] | -0.72 [-1.91; 0.52] | 0.02 [-1.08; 1.12] | | -0.48 [-1.59; 0.66] | |
| SRGS* | | -0.20 [-0.89; 0.49] | 0.01 [-1.14; 1.16] | 0.43 [-0.22; 1.07] | | 0.56 [-0.59; 1.67] | |
| CATS | | -0.14 [-0.94; 0.65] | -0.13 [-1.28; 1.02] | -0.45 [-1.32; 0.43] | | -0.13 [-1.22; 0.97] | |
| AUDIT-C | | 0.10 [-0.73; 0.93] | 0.09 [-1.07; 1.23] | -0.25 [-1.04; 0.54] | | 0.39 [-1.48; 0.74] | |
| BSSS* | | -0.01 [-1.05; 1.03] | -0.11 [-1.25; 1.05] | -0.22 [-1.35; 0.92] | | -0.17 [-1.26; 0.93] | |
| IUES | | 0.14 [-0.71; 1.00] | -0.23 [-1.38; 0.93] | 0.22 [-0.42; 0.86] | | 0.08 [-1.01; 1.17] | |
| BADS* | | 0.41 [-0.38; 1.20] | -0.84 [-2.05; 0.43] | 1.27 [0.07; 2.48] | | 0.13 [-0.97; 1.22] | |
| ATQ-R | | 0.65 [-0.10; 1.40] | 0.81 [-0.45; 2.02] | 0.14 [-0.82; -1.10] | | 0.41 [-0.72; 1.50] | |

**Table A.5** Results of linear regression models and Cohen’s d for efficacy outcomes at posttest (t1) and 6-month follow-up (t2) based on participants with clinically relevant symptoms of anxiety and/ or depression (subsample: N = 14; intervention group: n = 7; control group n = 7)

*Note.* CI= Confidence Interval; PHQ-ADS = Patient Health Questionnaire Anxiety and Depression Scale; CODI = Coping with a Disease; VAS = Visual Analogue Scale of the EuroQol Five-Dimensional Questionnaire- Youth; GSE = General perceived Self-Efficacy scale; SRGS = Stress-Related Growth Scale; CATS = Child and Adolescent Trauma Screen; AUDIT-C = Alcohol Use Disorders Identification Test (consumption items); BSSS = Berliner Social Support Scale (subscale: Actually received support, recipient); IUES = Internet Use Expectancies Scale; BADS = Behavioral Activation for Depression Scale; ATQ-R= Automatic Thoughts Questionnaire-Revised
* higher scores indicate better outcome; ^a^ controlling for baseline scores

|  | 12 weeks post-randomization (t1) | | 6 months post-randomization (t2) | |
| --- | --- | --- | --- | --- |
|  | Standardized regression coefficient  (95% CI)^a^ | Between-group effect size Cohens d (95% CI) | Standardized regression coefficient  (95% CI)^a^ | Between-group effect size Cohens d (95% CI) |
| **Outcome** |  |  |  |  |
| PHQ-ADS | -0.02 [-1.32; 1.29] | -0.04 [-1.17; 1.09] | -1.39 [-2.46; -0.33] | -2.48 [-4.50; -0.37] |
| CODI* | -0.29 [-1.21; 0.63] | -0.90 [-2.12; 0.39] | -0.01 [-0.48; 0.46] | -0.51 [-1.84; -0.25] |
| VAS* | 0.76 [-0.35; 1.87] | 0.64 [-0.58; 1.81] | 0.90 [-0.75; 2.55] | 1.05 [-0.49; 2.50] |
| GSE* | 0.46 [-0.33; 1.25) | -0.14 [-1.27; 1.00] | 0.35 [-1.02; 1.72] | -0.15 [-1.46; 1.18] |
| SRGS* | -0.29 [-1.41; 0.83] | 0.39 [-0.79; 1.53] | -0.88 [-3.83; 2.07] | 1.19 [-0.40; 2.67] |
| CATS | -0.54 [-1.73; 0.64] | -0.20 [-1.33; 0.94] | -1.56 [-2.55; -0.58] | -2.65 [-4.81; -0.40] |
| AUDIT-C | 0.10 [-0.39; 0.58] | 0.23 [-0.92; 1.36] | 0.49 [-0.96; 1.95] | 0.24 [-1.10; 1.55] |
| BSSS* | 0.31 [-0.92; 1.54] | 0.75 [-0.50; 1.93] | -0.49 [-2.33; 1.35] | -0.44 [-1.76; 0.93] |
| IUES | 0.03 [-1.21; 1.28] | 0.26 [-0.89; 1.39] | -1.01 [-2.12; 0.12] | -0.73 [-2.09; 0.71] |
| BADS* | -0.05 [-1.48; 1.39] | -0.32 [-1.45; 0.84] | 0.33 [-1.53; 2.19] | 0.37 [-0.99; 1.69] |
| ATQ-R | 0.02 [-1.40; 1.44] | 0.02 [-1.11; 1.15] | -0.82 [-2.80; 1.16] | -1.02 [-2.45; 0.51] |

**Table A.6** Results of linear regression models and Cohen’s d for efficacy outcomes at posttest (t1) and 6-month follow-up (t2) based on participants with no clinically relevant symptoms of anxiety and/ or depression (subsample: N = 14; intervention group: n = 6; control group n = 8)

*Note.* CI= Confidence Interval; PHQ-ADS = Patient Health Questionnaire Anxiety and Depression Scale; CODI = Coping with a Disease; VAS = Visual Analogue Scale of the EuroQol Five-Dimensional Questionnaire- Youth; GSE = General perceived Self-Efficacy scale; SRGS = Stress-Related Growth Scale; CATS = Child and Adolescent Trauma Screen; AUDIT-C = Alcohol Use Disorders Identification Test (consumption items); BSSS = Berliner Social Support Scale (subscale: Actually received support, recipient); IUES = Internet Use Expectancies Scale; BADS = Behavioral Activation for Depression Scale; ATQ-R= Automatic Thoughts Questionnaire-Revised
* higher scores indicate better outcome; ^a^ controlling for baseline scores

**Table A.7** Results of linear regression models and Cohen’s d for efficacy outcomes at posttest (t1) and 6-month follow-up (t2) based on per protocol analyses (subsample: N = 21; intervention group: n = 6; control group n = 15)

|  | 12 weeks post-randomization (t1) | | 6 months post-randomization (t2) | |
| --- | --- | --- | --- | --- |
|  | Standardized regression coefficient (95% CI) | Between-group effect size Cohens d (95% CI) | Standardized regression coefficient (95% CI) | Between-group effect size Cohens d (95% CI) |
| **Outcome** |  |  |  |  |
| PHQ-ADS | 0.07 [-0.78; 0.91] | 0.41 [-0.58; 1.38] | -0.52 [-1.33; 0.28] | -0.26 [-1.24; 0.73] |
| CODI* | -0.27 [-0.98; 0.43] | -0.41 [-1.38; 0.58] | 0.10 [-0.55; 0.76] | -0.02 [-1.00; 0.96] |
| VAS* | 0.37 [-0.54; 1.28] | 0.47 [-0.53; 1.44] | 0.54 [-0.50; 1.59] | 0.66 [-0.37; 1.66] |
| GSE* | 0.13 [-0.61; 0.87] | -0.40 [-1.37; 0.59] | 0.12 [-0.64; 0.89] | -0.32 [-1.30; 0.68] |
| SRGS* | -0.36 [-0.80; 0.08] | 0.27 [-0.70; 1.24] | 0.34 [-0.22; 0.89] | 1.20 [0.08; 2.27] |
| CATS | -0.08 [-0.65; 0.50] | 0.05 [-0.92; 1.02] | -0.82 [-0.62; -0.01] | -0.88 [-1.90; 0.19] |
| AUDIT-C | -0.01 [-0.54; 0.52] | -0.20 [-1.17; 0.77] | 0.06 [-0.79; 0.91] | -0.15 [-1.13; 0.84] |
| BSSS* | -0.22 [-1.01; 0.58] | -0.22 [-1.18; 0.76] | -0.36 [-1.39; 0.66] | -0.37 [-1.35; 0.63] |
| IUES | -0.00 [-0.80; 0.79] | -0.31 [-1.28; 0.67] | -0.11 [-0.80; 0.59] | -0.50 [-1.49; 0.51] |
| BADS* | -0.10 [-0.98; 0.79] | -0.56 [-1.54; 0.44] | 0.64 [-0.20; 1.48] | 0.20 [-0.79; 1.18] |
| ATQ-R | 0.60 [-0.11; 1.31] | 0.56 [-0.45; 1.54] | 0.11 [-0.69; 1.14] | -0.06 [-1.04; 0.92] |

*Note.* CI= Confidence Interval; PHQ-ADS = Patient Health Questionnaire Anxiety and Depression Scale; CODI = Coping with a Disease; VAS = Visual Analogue Scale of the EuroQol Five-Dimensional Questionnaire- Youth; GSE = General perceived Self-Efficacy scale; SRGS = Stress-Related Growth Scale; CATS = Child and Adolescent Trauma Screen; AUDIT-C = Alcohol Use Disorders Identification Test (consumption items); BSSS = Berliner Social Support Scale (subscale: Actually received support, recipient); IUES = Internet Use Expectancies Scale; BADS = Behavioral Activation for Depression Scale; ATQ-R= Automatic Thoughts Questionnaire-Revised
* higher scores indicate better outcome; ^a^ adjusted for baseline scores

**Table A.8** Means and standard deviations for the Intervention group and waiting list control group reported by caregivers

|  | Baseline (t0) | | | | 12 weeks post-randomization (t1) | | | | 6 months post-randomization (t2) | | | |
| --- | --- | --- | --- | --- | --- | --- | --- | --- | --- | --- | --- | --- |
|  | IG  *n* = 10 | | CG  *n* =10 | | IG *n = 10* | | CG  *n* = 9 | | IG  *n* = 10 | | CG  *n* = 9 | |
| Outcome | *M* | *SD* | *M* | *SD* | *M* | *SD* | *M* | *SD* | *M* | *SD* | *M* | *SD* |
| SMFQ | 4.50 | 6.19 | 2.40 | 3.06 | 6.30 | 6.53 | 5.78 | 6.13 | 5.30 | 6.95 | 4.78 | 3.63 |
| SCARED | 1.40 | 1.51 | 2.00 | 1.89 | 1.50 | 1.51 | 2.44 | 2.13 | 1.10 | 1.60 | 1.78 | 1.99 |
| CATS-C-D | 12.90 | 8.80 | 13.30 | 7.07 | 15.90 | 8.20 | 16.78 | 10.93 | 11.70 | 9.09 | 14.00 | 6.33 |
| BSSS* | 38.00 | 4.97 | 36.00 | 5.46 | 39.30 | 4.27 | 37.65 | 3.97 | 35.30 | 7.92 | 35.67 | 4.30 |

*Note.* M = Mean; SD = Standard deviation; IG = Intervention group; CG = Control group; SMFQ = Short version of the Mood and Feeling Questionnaire [score range: 0-26]; SCARED = Screen for Child Anxiety Related Emotional Disorders [score range: 0-10]; CATS-C-D = Child and Adolescents Trauma Screen-Caregiver [score range: 0-60]; BSSS = Berliner Social Support Scale [subscale: actually received support; score range: 11-44]
 *Higher score indicates better outcome

**Table A.9** Results of linear regression models and Cohen’s d for efficacy outcomes at posttest (t1) and 6-month follow-up (t2) reported by caregivers

|  | 12 weeks post-randomization (t1) | | 6 months post-randomization (t2) | |
| --- | --- | --- | --- | --- |
|  | Standardized regression coefficient  (95% CI) ^a^ | Between-group effect size Cohens d (95% CI) | Standardized regression coefficient  (95% CI)^a^ | Between-group effect size Cohens d (95% CI) |
| **Outcome** |  |  |  |  |
| SMFQ | -0.18 [-0.93; 0.58] | 0.09 [-0.82; 0.99] | -0.20 [-0.84; 0.44] | 0.10 [-0.81; 1.00] |
| SCARED | -0.30 [-1.16; 0.57] | -0.55 [-1.47; 0.41] | -0.20 [-1.14; 0.73] | -0.40 [-1.31; 0.53] |
| CATS-C-D | -0.02 [-0.82; 0.79] | -0.10 [-1.00; 0.81] | -0.15 [- 0.81; 0.50] | -0.26 [-1.16; 0.66] |
| BSSS* | 0.03 [0.70; 0.76] | 0.45 [-0.49; 1.36] | -0.18 [-1.23; 0.87] | -0.06 [-0.96; 0.84] |

*Note.* CI= Confidence Interval; SMFQ = Short version of the Mood and Feeling Questionnaire; SCARED = Screen for Child Anxiety Related Emotional Disorders; CATS-C-D = Child and Adolescents Trauma Screen-Caregiver; BSSS = Berliner Social Support Scale (subscale: actually received support)
*Higher score indicates better outcome; ^a^ controlling for baseline scores
